# 

**Published:** 1862-06

**Authors:** 


### BOOKS RECEIVED.

*Hand-Book of Surgical Operations; by* STEPHEN SMITH, M. D., *Surgeon to Bellevue Hospital. New York:* BAILLIERE BROTHERS, 440 *Broadway.*

*A Manual of Medical Diagnosis, being an Analysis of the Signs and Symptoms of Disease, by* A. W. BARCLAY, M. D., *Fellow of the Royal College of Physicians; Assistant Physician to St. George Hospital. Second American, from the second and revised London edition. Philadelphia:* BLANCHARD & LEA, 1862.

*The Surgical Adjuvant, and Reporter of Surgical Apparatus, Artificial Limbs, &c.; by* E. D. HUDSON, M. D., *(late Palmer & Co.) New York,* 1862.

*The Atlantic Monthly,* with the following table of contents: Walking—War and Literature—An order for a Picture—The South Breaker—The Sam. Adams Regiment in the town of Boston—Out of the body to God—The health of our Girls—Sonnet—The horrors of San Domingo—Methods of Study in Natural History—The Author of Charles Auchester—Astriæa at the Capitol—Pierre Antoine's Date Palm—"Solid Operations in Virginia"—Sunthin' in the Pastoral Line.

### ERIE COUNTY MEDICAL SOCIETY.

The semi-annual meeting of the Erie County Medical Society, will be held the second Tuesday in June, at the rooms of the Buffalo Medical Association, No. 7 South Division street. We desire to call attention to this meeting, and ask for a full attendance. There are physicians in the county every way eligible to membership, who have not yet complied with the statute "as made and provided," and are consequently not legally and strictly within the pale of the regular profession. We hope all such, will see the necessity and propriety of completing membership in this Society and will appreciate the advantages it will confer upon them.

